# Albumin and Neutrophil Combined Prognostic Grade as a New Prognostic Factor in Non-Small Cell Lung Cancer: Results from a Large Consecutive Cohort

**DOI:** 10.1371/journal.pone.0144663

**Published:** 2015-12-14

**Authors:** Haifeng Sun, Pingping Hu, Hongchang Shen, Wei Dong, Tiehong Zhang, Qi Liu, Jiajun Du

**Affiliations:** 1 Institute of Oncology, Shandong Provincial Hospital Affiliated to Shandong University, 324 Jingwu Road, Jinan, P.R. China; 2 Department of Thoracic Surgery, Shandong Provincial Hospital Affiliated to Shandong University, 324 Jingwu Road, Jinan, P.R. China; 3 Department of Radiation Oncology, Qianfoshan Hospital Affiliated to Shandong University, Jinan, Shandong, P.R. China; Peking University People Hospital, CHINA

## Abstract

**Objectives:**

It has been reported nutritional status and systemic inflammation were associated with the outcome of patients with malignancies. However, the prognostic value of combination of them was really scarce, especially in non-small cell lung cancer (NSCLC). In order to find a more simple and efficient predictor, we hypothesized that pretreatment albumin and neutrophil combined prognostic grade (ANPG) could offer an improved prognostic ability in NSCLC patients.

**Methods:**

We collected pretreatment albumin and neutrophil, clinicopathological, treatment and follow-up data of 1033 consecutive NSCLC patients treated between 2006 and 2011 in this retrospective study. The ANPG was calculated according to pretreatment albumin and neutrophil levels dichotomized by the optimal cut-off values, the quartile values and the clinical reference values. Kaplan-Meier (K-M) curves and Cox proportional regression were used for survival analyses. All the data was analyzed by SPSS 20.0.

**Results:**

According to optimal cut-off values and quartile values, significant differences were found in different pretreatment albumin, neutrophil levels and ANPG from the K-M curve (all *p*<0.05). Univariate analyses and multivariate analyses disclosed ANPG was a more sensitive independent predictor for both overall survival (OS) and progression free survival (PFS) than either albumin level or neutrophil level (HRs were higher for ANPG). As for clinical reference values, no significant difference of pretreatment albumin levels was found in K-M curve and univariate analyses. All three indexes lost their significance in multivariate analyses.

**Conclusion:**

Higher ANPG predicts worse OS and PFS in NSCLC patients independently, and it is more sensitive than hypoalbuminaemia and neutrophilia. It might be used as a reliable, convenient and more sensitive predictor to assist the identification of patients with poor prognosis and be a hierarchical factor in the future NSCLC clinical trials.

## Introduction

Lung cancer is the leading cause of malignancy-related mortality in both developed and developing countries, and non-small cell lung cancer (NSCLC) accounted for approximately 80% of all lung cancer deaths [1, 2]. Despite the tremendous development of technique, lung cancer remains dissatisfactory improvement in survival during the past decades [3]. To ameliorate the survival of NSCLC patients, specific and sensitive prognostic factors for identifying cancer risk which can provide a more appropriate estimation of individual outcomes and allow the optimization of patient stratification are desired in clinical trials.

Existing researches have certified that systemic inflammation has a close relationship with malignancies [4–7]. Besides, patients’ nutritional status is also closely linked to cancer mortality, with one third of deaths being caused by malnutrition rather than the cancer itself [8]. Thereafter, the predictive value for malignancies of combination of both systemic inflammation and nutritional status were increasingly investigated, such as Glasgow prognostic score (GPS) which defined as the combination of C-reactive protein (CRP) and albumin [9], Inflammatory-Nutritional Index (INI) which defined as ratio of albumin to CRP [10]. However, such kind of indexes were really scarce and dubious, and they were not tested routinely in clinical practice, especially in NSCLC.

As for systemic inflammation, cancer can induce local or systemic inflammation, which is mediated by activation of transcription factors and production of major inflammatory cytokines [11]. On the other hand, cancer-related inflammation can influence cell proliferation, cell survival, angiogenesis, tumor cell migration, invasion and metastasis of adaptive immunity [11]. Admittedly, neutrophil which is the most common and indispensable component of inflammation plays very important role in inflammatory tumor microenvironment [12]. Some studies have revealed derangement in the full blood count such as neutrophilia, was known poor independent prognostic factor for many solid tumors [13–15]. In addition, CRP which is the most widely accepted proxy for systemic inflammation was also identified as a prognostic factor in both advanced and resectable NSCLC [16, 17]. However, CRP is not commonly used in clinic because of low sensitivity and unconventional detection. Nutritional status of cancer patients, commonly reflected by serum albumin, is also a determinant of survival in many kinds of cancer. Hypoalbuminemia, an objective parameter of malnutrition [18], has been reported as a negative prognostic factor for survival in advanced NSCLC [19], and other malignancies [20, 21]. Nevertheless, the predictive efficiency of combination of pretreatment albumin level which reflects nutritional status and pretreatment neutrophil level which reflects systemic inflammation has not been reported before.

To find an index which can roundly and systematically reflect patients’ condition, we put albumin and neutrophil together to form a new index named albumin and neutrophil combined prognostic grade (ANPG). The aim of this study was to investigate and validate our hypothesis that ANPG might be a new, convenient and more powerful predictive factor for NSCLC patients.

## Patients and Methods

### Patient collection

A series of 1033 consecutive patients who underwent surgical treatment for primary NSCLC between January 2006 and September 2011 at the Department of Oncology of Shandong Provincial Hospital affiliated to Shandong University were retrospectively identified from the original electronic databases. Patient collection based on the following inclusion criteria: (1) patients who were pathologically diagnosed with NSCLC; (2) patients who underwent completely tumor resection; (3) patients who had complete serum albumin and neutrophil records before treatment within 1 week and (4) patients who had complete follow-up data. Patients were excluded who matched any of the following criteria: (1) patients who had ongoing non-cancer related inflammation, immunity disease or end-stage liver disease within 1 week; (2) patients who underwent previous chemotherapy and/or radiation therapy; (3) patients whose data were incomplete; (4) patients with previous or concomitant other cancers.

This research was approved by the Ethical Committee of Shandong Provincial Hospital affiliated to Shandong University and written informed consent was obtained by participants for their clinical records to be used in this study.

### Clinical and follow-up data collection

Clinical characteristics including gender, age, histological subtype, degree of tumor differentiation, pathological TNM stage (pTNM), postoperative radiotherapy and/or chemotherapy, pretreatment albumin and neutrophil count were recorded for all patients. After surgical treatment, all patients were regularly followed-up by phone interview. The pTNM was carried out according to the 8th edition of the TNM classification [22]. The degree of tumor differentiation was obtained from pathological report.

Progression free survival (PFS) was calculated from the time when firstly definite diagnosis to progression or death of any cause. Overall survival (OS) was measured from the date on which the first time definite diagnosis was made or the date of surgery until the date of death for any cause, the date of loss to follow-up or the date on which the patient was last known to be alive.

### Allocation of ANPG

Blood laboratory data, especially for albumin and neutrophil, was obtained before commencement of treatment. Three binary classification methods of pretreatment albumin and neutrophil were designed and the different cut-off points were as follows. (1) According to receiver operating characteristic (ROC) curve, the optimal cut-off values of pretreatment albumin and neutrophil were 42.55g/L and 2.895×10^9^/L not merely for OS but also for PFS, respectively. (2) The quartile values of pretreatment albumin and neutrophil were 43.80g/L and 3.070×10^9^/L, respectively. (3) The clinical reference values of pretreatment albumin and neutrophil were 35.00g/L and 7.000×10^9^/L, respectively.

The ANPG was calculated into 3 grades according to dichotomization of pretreatment albumin and neutrophil. Grade1 = elevated albumin and low neutrophil; grade2 = low albumin and low neutrophil, as well as elevated albumin and elevated neutrophil; grade3 = low albumin and elevated neutrophil.

### Statistical analyses

Statistical analyses were calculated by SPSS (version 20.0) software program (SPSS Inc., Chicago, IL, USA). Descriptive statistics were utilized to describe the characteristics of the study cohort. The optimal cut-off points were found out from ROC curve which is now widely recognized as the best approach for measuring the quality of diagnostic system. To ensure the best accuracy and diagnostic effect, the optimal cut-off value was located at the point on which the maximum absolute value [sensitivity-(1-specificity)] was calculated out. Kaplan-Meier (K-M) method was performed to determine the significance of variables for OS, PFS, and the log-rank test was utilized to examine the significant differences of survival distributions between different levels of albumin, neutrophil and ANPG. The Cox proportional hazards regression models were used for confirming the independent predictors of OS and PFS and multivariate Cox analyses were performed in a step-forward logistic regression approach. A two tailed *p*-value≤0.05 was considered to indicate a statistically significant difference.

## Results

### Patients’ characteristics

According to the inclusion criteria, a total of 1033 patients from the original files were finally retrospectively enrolled in our study. All samples were primary NSCLC patients who were pathologically diagnosed. Of these, the mean age was 59.18 years (range 20 to 83 years), and there were 745 (72.1%) patients ≤65years and 288 (27.9%) patients >65years, with 741 (71.7%) males and 292 (28.3%) females. Mean albumin was 41.3 (range from 25.2 to 48.3) g/L and mean neutrophil count was 4.28 (range from 1.01 to 12.32) ×10^9^/L. According to ROC curve, the number of patients with low and high level pretreatment albumin was 614 (59.4%) and 419 (40.6%), respectively; low and high level pretreatment neutrophil was 205 (19.8%) and 828 (80.2%), respectively; grade1, grade2 and grade3 of ANPG was 93 (9.0%), 438 (42.4%) and 502 (48.6%), respectively. According to quartile values, 782 (75.7%) were low albumin level and 251 (24.3%) were high; 259 (25.1%) were low neutrophil level and 774 (74.9%) were high; 75 (7.3%) achieved grade1, 360 (34.8%) achieved grade2 and 598 (57.9%) achieved grade3 for ANPG. According to clinical reference values, only 61 (5.9%) patients were low albumin level; only 68 (6.6%) patients were high neutrophil level and only 7 (0.7%) patients achieved grade3 for ANPG. All of the main baseline characteristics are detailed in Table 1.

**Table 1:** Baseline characteristics of all 1033 NSCLC patients.

	Characteristic		Data (%)
	No. of patients		1033 (100)
According to the optimal cut-off values	Pretreatment albumin level (mean±sd, g/L)		41.3±3.67
		Low	614 (59.4)
		High	419 (40.6)
	Pretreatment neutrophil level (mean±sd, 10^9^/L)		4.28±1.66
		Low	205 (19.8)
		High	828 (80.2)
	ANPG		
		Grade1	93 (9.0)
		Grade2	438 (42.4)
		Grade3	502 (48.6)
According to quartile values	Pretreatment albumin level		
		Low	782 (75.7)
		High	251 (24.3)
	Pretreatment neutrophil level		
		Low	259 (25.1)
		High	774 (74.9)
	ANPG		
		Grade1	75 (7.3)
		Grade2	360 (34.8)
		Grade3	598 (57.9)
According to clinical reference values	Pretreatment albumin level		
		Low	61 (5.9)
		High	972 (94.1)
	Pretreatment neutrophil level		
		Low	965 (93.4)
		High	68 (6.6)
	ANPG		
		Grade1	911 (88.2)
		Grade2	115 (11.1)
		Grade3	7 (0.7)
	Age (mean±sd, years)		59.18±9.69
		≤65years	745 (72.1)
		>65years	288 (27.9)
	Gender		
		Male	741 (71.7)
		Female	292 (28.3)
	Postoperative radio-chemotherapy		
		Yes	325 (31.5)
		No	708 (68.5)
	pTNM		
		Stage I,II	721 (69.8)
		Stage III	312 (30.2)
	Differentiation		
		Well or moderate	716 (69.3)
		Poor or undifferentiated	317 (30.7)
	Histological subtype		
		Adenocarcinoma	531 (51.4)
		Squamous cell	428 (41.4)
		Other	74 (7.2)
	OS (mean±sd, month)		44.29±23.63
	PFS (mean±sd, month)		36.47±26.72

Abbreviations: OS,overall survival; PFS, progression free survival; HR, hazard risk; CI, confidence interval; ANPG, pretreatment albumin and neuprophil combined prognostic grade; pTNM, pathological TNM stage; sd, standard deviation.

### Survival analyses in pretreatment albumin, neutrophil and ANPG

**Fig 1:**
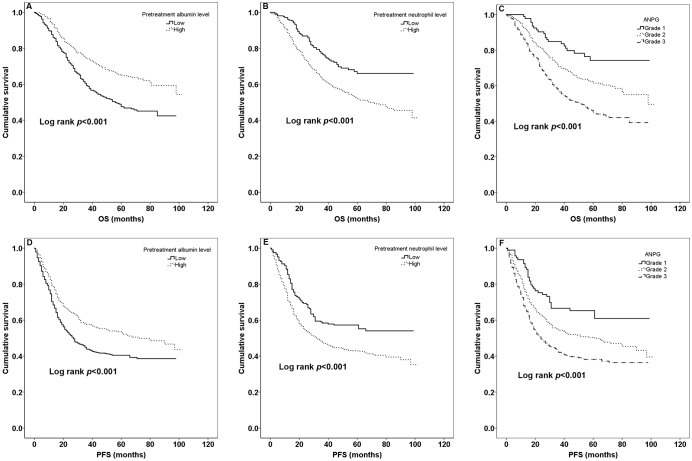
Kaplan-Meier curves for optimal cut-off values. The OS according to different pretreatment albumin levels, neutrophil levels and ANPG grades is shown in A, B, C, respectively. The PFS according to different pretreatment albumin levels, neutrophil levels and ANPG grades is shown in D, E, F, respectively.

**Fig 2:**
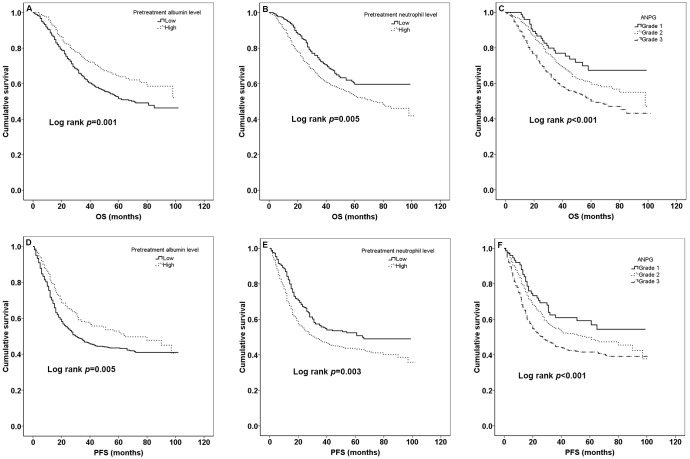
Kaplan-Meier curves for quartile values. The OS according to different pretreatment albumin levels, neutrophil levels and ANPG grades is shown in A, B, C, respectively. The PFS according to different pretreatment albumin levels, neutrophil levels and ANPG grades is shown in D, E, F, respectively.

**Fig 3:**
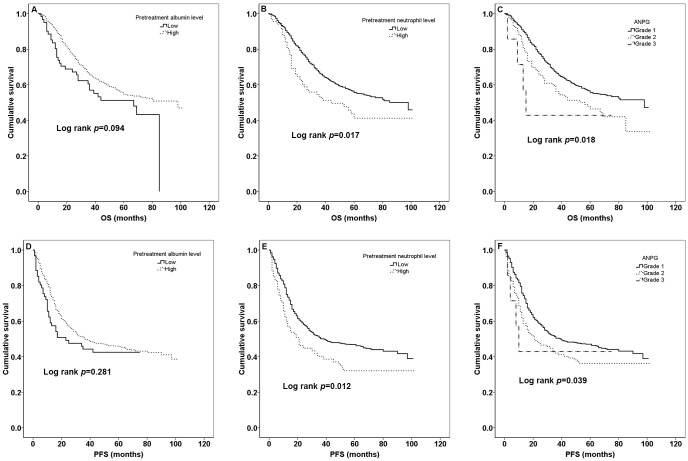
Kaplan-Meier curves for clinical reference values. The OS according to different pretreatment albumin levels, neutrophil levels and ANPG grades is shown in A, B, C, respectively. The PFS according to different pretreatment albumin levels, neutrophil levels and ANPG grades is shown in D, E, F, respectively.

The median PFS of all patients was 31 months [mean ± sd (standard deviation), 36.47±26.72] and the median OS was 45 months (mean ± sd, 44.29±23.63) during all patients’ follow-up period. 449 (43.5%) patients died and 563 (54.5%) patients made progression. K-M curves of pretreatment albumin, neutrophil levels and ANPG for OS and PFS according to optimal cut-off values, quartile values and clinical reference values were shown in Fig 1, Fig 2, Fig 3, respectively. Patients with high pretreatment albumin had a significantly better OS and PFS than low group (*p*<0.05) while it lost its significance for clinical cut-off determination. Moreover, OS and PFS rate for patients with high pretreatment neutrophil were worse than low group (all *p*<0.05). There was also a significant different survival in different ANPG grades both for OS and PFS (all *p*<0.05).

### Univariate survival analyses

All the results of univariate survival analyses were shown in Table 2.

**Table 2:** Univariate analyses.

			OS		PFS	
			HR (95% CI)	*p* value	HR (95% CI)	*p* value
According to the optimal cut-off values	Albumin					
		High level	1		1	
		Low level	1.699 (1.392–2.074)	<0.001	1.433 (1.205–1.704)	<0.001
	Neutrophil					
		Low level	1		1	
		High level	1.714 (1.313–2.238)	<0.001	1.511 (1.206–1.893)	<0.001
	ANPG					
		Grade 1	1		1	
		Grade 2	1.830 (1.162–2.882)	0.009	1.582 (1.103–2.270)	0.013
		Grade 3	2.987 (1.914–4.660)	<0.001	2.206 (1.548–3.145)	<0.001
According to quartile values	Albumin					
		High level	1		1	
		Low level	1.478 (1.171–1.864)	0.001	1.329 (1.088–1.625)	0.005
	Neutrophil					
		Low level	1		1	
		High level	1.384 (1.103–1.736)	0.005	1.341 (1.099–1.636)	0.004
	ANPG					
		Grade 1	1		1	
		Grade 2	1.409 (0.899–2.209)	0.135	1.287 (0.884–1.873)	0.188
		Grade 3	1.980 (1.283–3.056)	0.002	1.709 (1.189–2.455)	0.004
According to clinical reference values	Albumin					
		High level	1		1	
		Low level	1.356 (0.946–1.943)	0.097	1.204 (0.855–1.696)	0.287
	Neutrophil					
		Low level	1		1	
		High level	1.489 (1.068–2.077)	0.019	1.470 (1.084–1.993)	0.013
	ANPG					
		Grade 1	1		1	
		Grade 2	1.414 (1.081–1.849)	0.011	1.362 (1.063–1.744)	0.015
		Grade 3	1.911 (0.713–5.119)	0.198	1.399 (0.523–3.743)	0.504
	Postoperative radio-chemotherapy					
		No	1		1	
		Yes	1.535 (1.271–1.854)	<0.001	1.476 (1.245–1.749)	<0.001
	Gender					
		Female	1		1	
		Male	1.281 (1.035–1.586)	0.023	1.215 (1.007–1.466)	0.042
	Age					
		≤65years	1		1	
		>65years	1.360 (1.118–1.655)	0.002	1.195 (0.999–1.430)	0.051
	pTNM					
		Stage I,II	1		1	
		Stage III	3.148 (2.611–3.796)	<0.001	2.360 (1.994–2.794)	<0.001
	Differentiation					
		well or moderate	1		1	
		poor or undifferentiated	1.420 (1.171–1.721)	<0.001	1.319 (1.108–1.571)	0.002
	Histological subtype					
		Adenocarcinoma	1		1	
		Squamous cell	1.248 (1.031–1.512)	0.023	1.054 (0.888–1.252)	0.546
		Other	1.025 (0.695–1.511)	0.903	0.861 (0.606–1.223)	0.403

Abbreviations: OS,overall survival; PFS, progression free survival; HR, hazard risk; CI, confidence interval; ANPG, pretreatment albumin and neuprophil combined prognostic grade; pTNM, pathological TNM stage.

As for ROC cut-off determination, low pretreatment albumin level [hazard ratio (HR) = 1.699, 95% confidence interval (CI): 1.392–2.074, *p*<0.001], high pretreatment neutrophil level (HR = 1.714, 95%CI: 1.313–2.238, *p*<0.001), high ANPG (HR = 1.830, 95%CI: 1.162–2.882, *p* = 0.009, grade2/grade1; HR = 2.987, 95%CI: 1.914–4.660, *p*<0.001, grade3/grade1) were associated with worse OS. As for quartile cut-off determination, low albumin level (HR = 1.478, 95%CI: 1.171–1.864, *p* = 0.001), high neutrophil level (HR = 1.384, 95%CI: 1.103–1.736, *p* = 0.005), high ANPG (HR = 1.409, 95%CI: 0.899–2.209, *p* = 0.135, grade2/grade1; HR = 1.980, 95%CI: 1.283–3.056, *p* = 0.002, grade3/grade1) were also proved to be poor outcome factors for OS. As for clinical cut-off determination, only high neutrophil level (HR = 1.489, 95%CI: 1.068–2.077, *p* = 0.019) and grade2 of ANPG (HR = 1.414, 95%CI: 1.081–1.849, *p* = 0.011, grade2/grade1) retained significance for OS. In the analyses about PFS, the HRs for ANPG were also generally higher than pretreatment albumin and neutrophil levels according ROC and quartile cut-off determinations, implying more important prognostic value. Other identified prognostic factors for OS and PFS including postoperative radio-chemotherapy, gender, age, pTNM, degree of tumor differentiation (all *p*<0.05). Of note, age>65years was only a nearly univariate prognostic predictor for PFS (*p* = 0.051) and histological subtype only significantly predicted for OS (HR = 1.248, 95%CI: 1.031–1.512, *p* = 0.023, squamous cell/adenocarcinoma).

### Multivariate survival analyses

The multivariate Cox proportional regression in which variables were tested in a step-forward logistic regression approach was performed to examine independent factors for OS and PFS. Pretreatment albumin level, pretreatment neutrophil level and ANPG were respectively brought into the model with all other significant factors in univariate survival analyses. Results of the three multivariate survival analyses were successively shown in Table 3.

**Table 3:** Multivariate analyses.

		According to the optimal cut-off values				According to quartile values			
		OS		PFS		OS		PFS	
		HR (95% CI)	*p* value	HR (95% CI)	*p* value	HR (95% CI)	*p* value	HR (95% CI)	*p* value
Albumin									
	High level	1		1		1		1	
	Low level	1.645 (1.347–2.009)	<0.001	1.417 (1.191–1.687)	<0.001	1.325 (1.048–1.675)	0.019	1.259 (1.028–1.541)	0.026
Age									
	≤65years	1		1		1		1	
	>65years	1.601 (1.310–1.958)	<0.001	1.366 (1.111–1.605)	0.002	1.605 (1.311–1.963)	<0.001	1.353 (1.125–1.626)	0.001
pTNM									
	Stage I,II	1		1		1		1	
	Stage III	3.260 (2.680–3.966)	<0.001	2.367 (1.984–2.823)	<0.001	3.403 (2.812–4.119)	<0.001	2.350 (1.970–2.803)	<0.001
Postoperative radio-chemotherapy									
	No	1		1		*		1	
	Yes	1.229 (1.011–1.493)	0.038	1.249 (1.048–1.488)	0.013	*		1.245 (1.045–1.484)	0.014
Gender									
	Female	*		*		1		*	
	Male	*		*		1.278 (1.031–1.583)	0.025	*	
Neutrophil									
	Low level	1		1		1		1	
	High level	1.639 (1.255–2.141)	<0.001	1.439 (1.147–1.804)	0.002	1.349 (1.076–1.692)	0.01	1.297 (1.062–1.583)	0.011
Age									
	≤65years	1		1		1		1	
	>65years	1.639 (1.324–2.001)	<0.001	1.373 (1.143–1.648)	0.001	1.665 (1.364–2.033)	<0.001	1.386 (1.154–1.663)	<0.001
pTNM									
	Stage I,II	1		1		1		1	
	Stage III	3.390 (2.802–4.101)	<0.001	2.365 (1.983–2.821)	<0.001	3.403 (2.813–4.117)	<0.001	2.366 (1.985–2.822)	<0.001
Postoperative radio-chemotherapy									
	No	*		1		*		1	
	Yes	*		1.213 (1.017–1.445)	0.031	*		1.225 (1.028–1.459)	0.023
ANPG									
	Grade 1	1		1		1		1	
	Grade 2	1.771 (1.124–2.792)	0.014	1.493 (1.039–2.145)	0.03	1.538 (0.980–2.413)	0.061	1.323 (0.908–1.928)	0.145
	Grade 3	2.790 (1.786–4.359)	<0.001	2.065 (1.445–2.950)	<0.001	1.922 (1.244–2.969)	0.003	1.638 (1.139–2.357)	0.008
Age									
	≤65years	1		1		1		1	
	>65years	1.570 (1.284–1.919)	<0.001	1.318 (1.097–1.584)	0.003	1.165 (1.321–1.974)	<0.001	1.346 (1.120–1.617)	0.002
pTNM									
	Stage I,II	1		1		1		1	
	Stage III	3.385 (2.797–4.095)	<0.001	2.364 (1.982–2.820)	<0.001	3.381 (2.793–4.094)	<0.001	2.349 (1.969–2.803)	<0.001
Postoperative radio-chemotherapy									
	No	*		1		*		1	
	Yes	*		1.219 (1.022–1.453)	0.027	*		1.227 (1.030–1.462)	0.022

Abbreviations: OS,overall survival; PFS, progression free survival; HR, hazard risk; CI, confidence interval; ANPG, pretreatment albumin and neuprophil combined prognostic grade; pTNM, pathological TNM stage.

* Not in the final step of multivariate analyses.

As for ROC cut-off determination, pretreatment albumin level, pretreatment neutrophil level, ANPG were all significantly independent prognostic factors (*p*<0.05) and ANPG [HR = 1.771(grade2/grade1), HR = 2.790(grade3/grade1) for OS; HR = 1.493(grade2/grade1), HR = 2.065(grade3/grade1) for PFS] presented higher HR than albumin (HR = 1.645 for OS; HR = 1.417 for PFS) and neutrophil (HR = 1.639 for OS; HR = 1.439 for PFS). Age and pTNM were also independently prognostic for OS and PFS (all *p*<0.05). Noteworthily, postoperative radio-chemotherapy was also an independent predictor but it had no significant impact on OS when it was analyzed with neutrophil or ANPG.

As for quartile cut-off determination, pretreatment albumin level, pretreatment neutrophil level, ANPG (grade3/grade1) were also significantly independent prognostic factors (*p*<0.05). ANPG [HR = 1.538(grade2/grade1), HR = 1.922(grade3/grade1) for OS; HR = 1.323(grade2/grade1), HR = 1.638(grade3/grade1) for PFS] still presented higher HR than albumin (HR = 1.325 for OS; HR = 1.259 for PFS) and neutrophil (HR = 1.349 for OS; HR = 1.297 for PFS). Age and pTNM were also independently prognostic for OS and PFS (all *p*<0.05). Besides, postoperative radio-chemotherapy only had significantly independent impact on PFS instead of OS. Gender was also proved to be an independent predictor for OS when it was analyzed with pretreatment albumin level (*p* = 0.025).

Pretreatment albumin level, pretreatment neutrophil level and ANPG were not included in the final step of multivariate analyses according to clinical cut-off determination (not shown in Table 3).

## Discussion

Since high mortality and dissatisfactory improvement of lung cancer, the major prognostic factors have been searched all the time and numerous clinical indexes were identified to be significantly related to lung cancer survival. It has been reported systemic inflammation and nutritional status were closely related to NSCLC [7, 23]. The connection between inflammation and survival of NSCLC dates back to early of 21 century [24]. After decade years, mounting reports have provided solid evidence to prove prognostic value of systemic inflammation and nutritional status which can be easily quantified and reflected by peripheral neutrophil and serum albumin [13, 19]. In our study, we firstly took albumin and neutrophil together to evaluate whether the combination of them could present a better predictive value for NSCLC patients’ survival. Strikingly, we found ANPG not only was a strong independent predictor but also had a higher sensitivity than either of them.

Neutrophil, an important component of inflammation, is the first type of immune cell that responds to the site of infection and attack invaders directly. Actually, neutrophil guards its conventional positive character as a defender by killing not only invading pathogens but also malignancies. However, the inflammatory cells and cytokines found in tumors are more likely to contribute to tumor growth, progression, and immunosuppression than they are to mount an effective host antitumor response [25]. In 1986, Shoenfeld and colleagues found that high peripheral blood leukocyte count indicated worse prognosis in patients with non-hematologic neoplasms [26]. Thereafter, an increasing number of studies demonstrated neutrophil was related to poor outcome in multiple tumors [27], also in NSCLC [13]. These early reports were consistent with our findings in this study that high pretreatment neutrophil level was associated with worse survival in NSCLC patients. Although exact mechanism for this remains unclear, the reason may be that neutrophils can be recruited by kinds of chemoattractant mediators into tumor microenvironment then become pro-tumor N2 phenotype tumor-infiltrating neutrophil (N2-TIN) which can improve tumor progression [12, 28]. Albumin, which is produced by liver, is usually regarded as an index of malnutrition and cachexia when decreased. Evidence suggested that inflammation could suppress albumin synthesis [29] and the progressive decrease of albumin was a consequence of systemic inflammation [30]. As another inflammatory index, hypoalbuminemia was also reported to be associated with poor survival in NSCLC [19]. We speculated the predict value of low pretreatment albumin might be due to dystrophic, lack of immunity and weak status of body. Moreover, chemotherapeutics may remain high residue and high toxicity in the blood stream because of lacking of albumin to bind to the drugs and this may also contribute to cancer mortality. However, both of pretreatment albumin and neutrophil levels have not been commonly used in clinic because of their low predict efficiency. The volatility of albumin also limits its application in clinic. Therefore, we gave a hypothesis here that taking pretreatment albumin and neutrophil together, ANPG would be a more powerful and feasible predictor for NSCLC patients. Additionally, other researches referring to this combined prognosis score is really scarce, especially for NSCLC.

In this study, we firstly explored the prognostic value of the combination of serum albumin and neutrophil in NSCLC patients. Our results suggested that when adopting ROC and quartile cut-off determinations for dichotomy of albumin and neutrophil, patients with low pretreatment albumin, elevated pretreatment neutrophil, high ANPG grade had worse OS and PFS in certain extent. Although all three of them were independently related to poor survival outcomes, ANPG offered higher HRs than the two other indexes, which supported our hypothesis that ANPG presented more powerful prognostic value in univariate and multivariate analyses.

When using clinical cut-off determination, pretreatment albumin lost its significance on predicting outcome for NSCLC, and ANPG was also disqualified for independent prognostic factor in multivariate analyses. This might be due to serious maldistribution of dichotomy and tiny sample size of low albumin level, high neutrophil level and grade3 (ANPG). Besides, for patients initially diagnosed as primary lung cancer, most haematoglgical parameters of them had not changed too much and were still in normal clinical reference range. Therefore, the clinical reference cut-off values may not be suitable for dichotomy of these patients. Even so, K-M curve and survival data indicated that there still existed worse prognostic trend for patients with low pretreatment level, high pretreatment level and high ANPG grade (Fig 3).

Noteworthily, haematological tests are one of the routine kinds of tests carried out in cancer patients, and the ANPG will be easily obtained in clinic. Compared with existing factors, our study might provide a new, highly reproducible, easily obtainable, low cost and most of all more powerful index for predicting outcome and making therapeutic decisions. Even so, the authentic value of ANPG should be confirmed in clinic. Moreover, what we have discovered did not clarify precise mechanism underlying the relationship between this combined index and NSCLC prognosis. Further studies are required to address this question.

## Conclusion

Our study firstly established a new index named ANPG for predicting outcome of primary NSCLC after surgical treatment by putting pretreatment albumin and neutrophil together. We not only demonstrated ANPG was an significantly independent prognostic factor for NSCLC patients and patients with high ANPG seemed to have more chance to live in poor prognosis, but also found ANPG outperformed better than either albumin or neutrophil for predicting outcome and making therapeutic decisions for NSCLC. However, the potential mechanisms and performance for clinical practice should be validated in further prospective studies.
